# Microfluidic Synthesis of Scalable Layer-by-Layer Multiple Antigen Nano-Delivery Platform for SARS-CoV-2 Vaccines

**DOI:** 10.3390/vaccines12030339

**Published:** 2024-03-21

**Authors:** Yang Xu, Kazuya Masuda, Christine Groso, Rick Hassan, Ziyou Zhou, Kelsey Broderick, Moriya Tsuji, Christopher Tison

**Affiliations:** 1 Luna Labs USA, LLC, Charlottesville, VA 22903, USAkelsey.r.broderick@gmail.com (K.B.); chris.tison@lunalabs.us (C.T.); 2 Aaron Diamond AIDS Research Center, Division of Infectious Diseases, Department of Medicine, Columbia University Irving Medical Center, New York, NY 10032, USA; km3466@cumc.columbia.edu (K.M.); cg3487@cumc.columbia.edu (C.G.);

**Keywords:** layer-by-layer (LbL), chitosan nanoparticle, SARS-CoV-2, T-cells, sustained releases, adjuvant

## Abstract

The COVID-19 outbreak was a global pandemic with wide-ranging healthcare implications. Although several mRNA-based vaccines delivered using lipid nanoparticles (LNP) have been approved and demonstrated efficacy at reducing the severity and spread of infection, continued rapid viral evolution and disadvantages currently associated with LNP delivery vehicles (such as toxicity) are driving the design of next-generation SARS-CoV-2 vaccines. Herein, we describe the development of a trimethylated chitosan-based nanoparticle layer-by-layer (LbL) delivery platform for multiple antigens as a scalable and safe COVID-19 vaccine, known as, “LbL-CoV19”. These vaccine candidates have been demonstrated to be biocompatible, safe, and effective at stimulating both humoral and cellular responses for protection in preclinical studies. Preliminary results also indicate that LbL-CoV19 can potentially achieve rapid, long-lasting, and broad protection against the SARS-CoV-2 challenge. The “plug-and-play” platform technology is well suited to preparedness for future pandemics and disease outbreaks.

## 1. Introduction

The emergence of severe acute respiratory syndrome coronavirus 2 (SARS-CoV-2) in 2019 caused a global pandemic, and over the last half-decade, several coronavirus vaccines have received authorization for use from the United States Food and Drug Administration (FDA). Though these vaccines have been approved and have successfully demonstrated a reduction in the severity and spread of infection, continued rapid mutation of the virus suggests that a safer and shelf-stable vaccine formulation using a plug-and-play delivery platform with composition controllability, high loading efficiency, and low price, is still needed for this and future pandemic threats.

Subunit vaccines are one potential option besides Pfizer’s or Moderna’s current nucleic acid-based vaccines, as they maintain an improved safety profile compared to inactivated vaccines [1]. However, the immunogenicity of these vaccines is usually low, and thus they often require the inclusion of adjuvants or delivery platforms to enhance the biological half-life of the antigenic material or improve the immunomodulatory cytokine response [2]. Furthermore, a clinical trial survey indicated that most developmental vaccine candidates have used the structural S-protein (SP) of SARS-CoV-2 as the target since SP is considered the most suitable antigen to induce neutralizing antibodies. This includes the current FDA-authorized Novavax COVID-19 vaccine [3]. However, COVID-19 vaccine candidates should not be limited to SP. Investigating the entire proteome of SARS-CoV-2 will likely identify other non-structural proteins (NSP) or open-reading frame (ORF) accessory proteins [4] that are crucial to the pathogenicity of the virus, viral adhesion, replication, and host invasion [5]. Clinical results demonstrated that 10 out of 175 patients who recovered from COVID-19 were seronegative, with no antibodies produced [6]. This suggests that factors other than antibodies, such as T-cells and cytokine response, may contribute to protecting these seronegative patients [7]. As a result, the ideal next-generation vaccine or vaccine platform must be carefully designed to induce broadly neutralizing antibody and cellular responses to achieve full and long-lasting protection [8]. 

Here, we describe the development of trimethylated chitosan (TMC)-based nanovaccine delivery platform that is synthesized using a scalable microfluidic system. This layer-by-layer (LbL) synthetic platform permits codelivery of both S-protein/peptide (SP) and non-structural or accessory protein/T-cell epitope peptides of SARS-CoV-2, while also providing for the addition of an adjuvant in the delivery complex. Chitosan, a special cationic polysaccharide, is a product extracted from the deacetylation of chitin. It has numerous advantages for vaccine and drug delivery, such as nontoxicity, biocompatibility, biodegradability, strong mucoadhesive properties, and good cost performance [9]. This LbL trimethylated chitosan-based delivery platform could enhance antigen stability, prolong the duration of action, control drug release, optimize dissolution of poorly soluble peptides, and increase the cell membrane permeability of hydrophobic antigens such as peptides [10]. Conventional methods for the production of chitosan nanoparticles have been developed in several research groups [11,12], but none of them has been used in the clinic. Recently, multiple antigen delivery using a trimethylated chitosan-based layer-by-layer delivery vehicle has been demonstrated in our lab for malaria vaccination, as further described in a recent publication [13]. However, scaled production to meet the requirements of potential vaccine rollout was determined to be a remaining challenge for chitosan-based nanoparticle platforms. Most of the reported approaches described for the synthesis of chitosan-based nanoparticles are not suitable for large-scale synthesis. Spray drying and ionic gelation methods could potentially be employed for the large-scale synthesis of chitosan nanoparticles, but particle size arising from these production methods shows a greater distribution between 160 nm and 1 µm [14].

**Figure 1:**
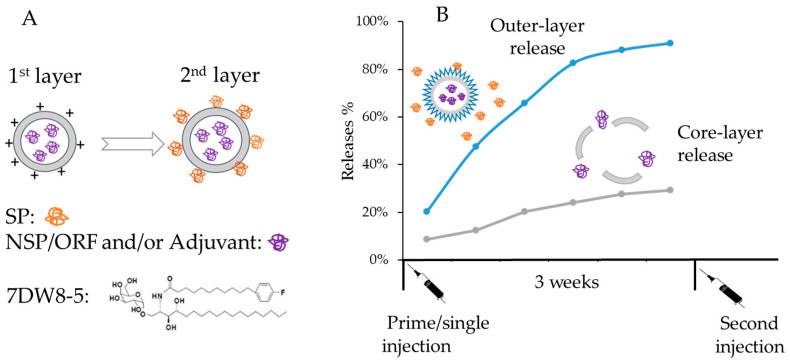
Scalable plug-and-play Layer-by-Layer (LbL) trimethylated chitosan NP platform for loading of SARS-CoV-2 antigens including Spike protein/peptide (SP) and Non-structural protein/peptide (NSP) as a novel vaccine candidate, (LbL-CoV19). (**A**) The loaded antigens in each layer could be one or two antigens or open-reading frame accessory protein/peptides (ORF) or one antigen with an adjuvant 7DW8-5; (**B**) The LBL sustained releases profile of each antigen has been demonstrated in vaccination for potential long-term protection.

Here, we report a microfluidic-based method that can be adopted for the larger-scale production of LbL trimethylated chitosan (TMC)-NP-based vaccine candidates (LbL-CoV19). Inside the core of the NP, the peptide or an adjuvant can be encapsulated, while the SP can be adsorbed to the outer layer (Schematic Figure 1) for use as the vaccine candidates. The adjuvant 7DW8-5 was developed in our partner Dr. Tsuji’s lab, and it has several critical differences from α-GalCer, with a fluorinated benzene ring at the end of the C8-length fatty acyl chain. 7DW8-5 demonstrated stronger bioactivity toward *i*NKT cells and CD1d-bearing DCs than α-GalCer. It has been demonstrated that 7DW8-5 significantly increased (up to 9-fold) CD8^+^ T cell responses after both priming and boosting phases while showing no systemic reactogenicity [15]. In the LbL-COV19 vaccine candidate, after intramuscular or subcutaneous administration, SP is first released to provide immediate protection from virus entry, and subsequently, NSP/adjuvant is released for enhancement of cellular/T cell responses and inhibition of viral replication. Further, this platform is designed to allow “plug-and-play” modifications with different antigens from virus variants or T-cell epitope peptides for enhanced preparedness for future disease outbreaks.

## 2. Materials and Methods

### 2.1. Materials

Chitosan (75–85% deacetylated, mol wt. 50–190 kDa) and sodium tripolyphosphate pentabasic (TPP, ≥98.0%, Cat. No. 72,061) were purchased from Sigma-Aldrich (St. Louis, MO, USA). Spike protein (SARS-CoV-2 Spike protein S1, Omicron Variant, Cat. No. Z03729) was purchased from GenScript (Piscataway, NJ, USA), and peptides or dye-labeled peptides were custom synthesized by GenScript (Piscataway, NJ, USA).

### 2.2. Trimethlyated Chitosan Nanoparticle Synthesis, and LbL-CoV19 Formulation Synthesis, Loading, and Release Tests

#### 2.2.1. Trimethlyated Chitosan Nanoparticle Synthesis Using a Microfluidic Device

A microfluidic nanoparticle synthesis system (NanoGenerator^TM^ Flex, PreciGenome, San Jose, CA, USA) including a PG-MFC pressure controller was used for Trimethylated chitosan (TMC) nanoparticle production. The UI Advanced mode Flow control software (v01.21 210916) was integrated for automation. The flow rate from each reservoir was controlled by a flow sensor. A Micro-mixer chip (CHP-MIX-3, PreciGenome) was used for achieving the rapid, highly efficient, and controlled mixing of different precursor solutions or phases. For nanoparticle production, TMC precursors were produced using our recently published method [13]. The zeta potential of the TMC solution was measured before use to ensure it was in the correct range (40–50 mV) for formulation synthesis. The TMC solution was filled in Reservoir 1 at a concentration between 1 and 5 mg/mL. TPP solution was filled in Reservoir 2 at a concentration of 1 mg/mL. During experiments, pre-set pressures from the PG-MFC pressure controller were applied to each reservoir. Solutions in each reservoir were pushed through tubing into the two inlets of a micro-mixer chip and mixed inside the channel of the microfluidic chip. The consumed TMC solution was 1 mL and the consumed TPP solution was 0.2 mL when the ratio of TMC and TPP was set at 5:1, and the obtained NP solution was approximately 1.2 mL. The total reaction time for this volume was 12 s. Different ratios of TMC and TPP were tested based on previously developed NP synthesis precursor feed ratios (10:1–3:1) to determine the optimal synthesis conditions. To obtain a large scale of NP, we filled the precursor solution in the reservoirs with a large volume and set the multiple runs of the reactions to collect the required volume of NP solution.

#### 2.2.2. LbL-CoV19 Formulation Preparation Using a Microfluidic Device

TMC was dissolved in ultrapure DI water at a concentration of 1.5 mg/mL and TPP was dissolved in ultrapure DI water at a concentration of 2 mg/mL. A volume of 50 μL of Spike protein or peptide (antigen) solution at a concentration of 40 mg/mL was added into the TMC solution; the concentration of antigen was 0.3 mg/mL, and the ratio of TMC to antigen was 5:1. After mixing, the TMC/antigen solution was placed in a 15 mL FALCON tube, which could be fit into a 2-port connector as Reservoir 1 in the microfluidic system. The TPP solution was put into the FALCON tube as Reservoir 2. The flow rate of TMC was set at 5 mL/min, the flow rate of TPP was set at 1 mL/min, and the total flow rate was 6 mL/min. The consumed TMC solution was 1 mL and the consumed TPP solution was 0.2 mL for each reaction. The ratio of TMC and TPP was 5:1 and the total obtained TMC-Spike-TPP solution was approximately 1.2 mL. The reaction time was 0.2 min (12 s). After the reaction was complete, samples were evaluated by DLS for size distribution. The same method was used for other peptide encapsulation; the one-layer or two-layer formulation had the TMC:TPP: Spike or TMC:TPP:peptide: Spike components at final mass ratios of 5:1:1 or 5:1:0.5:1, respectively. 

#### 2.2.3. LbL-CoV19 Formulation Release Test

First, TMC was dissolved in ultrapure DI water at a concentration of 1 mg/mL. Separately, tripolyphosphate (TPP) was dissolved in DI water at a concentration of 1 mg/mL. FTIC dye-labeled peptide was dissolved in DMSO at a concentration of 40 mg/mL. His-tagged SARS-CoV-2 spike protein was dispersed in DI water at a concentration of 1 mg/mL. A volume of 1 mL of TMC solution (1 mg/mL, 1 mg) was mixed with 5 µL of peptide solution (0.2 mg) and stirred (~200 rpm) for 15 min. A volume of 0.2 mL of TPP solution (1 mg/mL, 0.2 mg) was added dropwise into the TMC/Peptide solution under stirring. After 1 h, DLS and zeta potential measurements were conducted. Finally, spike protein solution (0.2 mg) was added into the TMC/Peptide/TPP solution by magnetic stirring for 1 h. After the reaction, the final sample was evaluated by DLS and zeta potential. In addition to the above procedure, we also investigated adding a purification step before constructing the spike protein on the second layer by centrifugation filtration using a 50 K cut-off filter. This second method (labeled “purification”) was compared against the original process that did not involve the purification step (labeled “no purification”).

The formulations were placed in 300 kDa molecular-weight cutoff (MWCO) filtration tubes. PBS was added to the top of the MWCO 300 kDa tubes and filled to the edge. The tubes were incubated in a shaker (200 rpm) at 37 °C. After incubation for varying times (0, 1 h, 4 h, 1 day, 7 days, 14 days, and 21 days), tubes were centrifuged at 5000 rpm for 10 min. The bottom solution was collected, labeled, and stored in the fridge before ELISA testing for quantification of released Spike protein or HPLC-MS for quantification of the released peptide. At each time point, 0.5–0.7 mL was collected for evaluation and additional fresh PBS solution was added to the top of the tubes before continued incubation. Standard curves were generated for both the ELISA and HPLC-MS methods for the calculation of antigen concentration. 

#### 2.2.4. LbL-CoV19 Formulation Lyophilization

Lyophilization transforms the LbL-CoV19 formulation from a liquid to a stable solid, which can extend the shelf life of vaccine candidates. First, formulation solutions are frozen at −20 °C overnight in a 10 mL or 50 mL glass vial. The formulation was then directly loaded into the lyophilizer (FreeZone 6, LABCONCO, Kansas City, MI, USA). The FreeZone was programmed for 3 segments as follows. Segment 1: Temperature decrease to −40 °C at a rate of −1.5 °C/min. Hold the temperature for 3 h once attained. Segment 2: Hold −40 °C for 24 h while under vacuum. Segment 3: Temperature increases to 25 °C at a rate of 1 °C/min. Once −40 °C was attained during Segment 1, the frozen samples were transferred to the lyophilization chamber. The lyophilization chamber was then put under vacuum, and the program was allowed to continue as described. During Segment 3, the samples were examined to ensure complete drying had occurred, at which point the samples were removed from the lyophilizer and immediately capped to be placed at a 4 °C refrigerator for storage.

#### 2.2.5. Scanning Electron Microscopy Evaluation of TMC NPs and LbL-CoV19 Formulation

Lyophilized nanoparticles were resuspended at 1 mg/mL in ethanol by gentle vortexing. Once it was dispersed uniformly in solution, 10 µL was pipetted onto a chromium-coated silica wafer adhered to an SEM stub with carbon tape. After drying for 24 h in a desiccator, stubs were placed within an electron beam coater (Leica EM ACE600, Deerfield, IL, USA) and sputter coated with approximately 12 nm of gold–platinum and then placed within the SEM chamber (Zeiss Sigma VP HD field, White Plains, NY, USA). Images of the TMC NPs and LbL-CoV19 formulations were taken at 100×, 500×, 5000×, 10,000×, and 30,000× with triplicate images taken at 10,000 and 30,000× using a ZEISS scanning electron microscopy for quantitative measurements. Image analysis of the nanoparticles was completed on the 30 kX images with the in-line tool in Image J (Version 1.54). N = 20 measurements were taken per image, with N = 3 images of each sample, for a total of 60 measurements obtained from each sample.

- Dynamic Light Scattering (DLS) and Zeta potential analysis of TMC nanoparticles and LbL-CoV19 formulations

The DLS size and zeta potential (surface charge) of the TMC nanoparticles or LbL-CoV19 formulation nanoparticles were measured with a Zetasizer (Nano 139 ZS90, Malvern Panalytical, Westborough, MA, USA) at each LbL synthesis step. The particle-size distribution is reported as a polydispersity index (PDI). Each formulation solution was placed in a cell with no air bubbles in the instrument holder. The nanoparticle solution was characterized using standard procedures following Malvern Instruments’ manual guidelines. All measurements were performed with 3–5 replicates and presented as average ± standard deviation.

- ELISA method for spike protein identification

For the recombinant spike protein containing the receptor binding domain (RBD) and a His-tag, His-tag ELISA Detection Kit (Cat.L00436, GenScript), a competitive ELISA quantification method for evaluation of the loading and release of the spike protein. Briefly, following sample preparation, the samples were tested in triplicate by performing the ELISA for spike protein according to the manufacturer’s protocol. For this assay, spike protein was serially diluted in the assay diluent provided in the kit and distributed to a round bottom microplate (clear, 50 µL/well) coated with a His-tagged protein (12.7 kDa). Anti-His Monoclonal Antibody (A00186, GenScript, 50 µL/well) was added, and the plate was covered and incubated for 30 min at RT. Following protein competition with antibodies, the solution was removed, and the plate was washed 4 times with 260 µL of wash solution. Antibody Tracer (100 µL/well) was added and incubated for an additional 30 min at RT. The plate was again washed 4 times with 260 µL of wash solution. TMB substrate (100 µL/well) was added and incubated for an additional 10 min at RT, with the subsequent addition of 50 µL of the Stop Solution and measurement of the absorbance at 450 nm. Absorbance values were plotted in Excel and a linear trendline was applied for spike protein quantifications.

- HPLC-MS method for peptide identification

High-performance liquid chromatography–mass spectrometry (HPLC-MS) was utilized for peptide identification and quantification as a quality control method. The released peptide was identified by the retention time and mass-to-charge ratio (*m*/*z*). For all analyses, a C18 column (Inertsil ODs-3, 4.6 × 250 mm) was used along with a Diode Array Detector (DAD) set to collect data at 220 nm in an HPLC (Agilent 1100) instrument. In direct sequence with the HPLC, the Agilent mass spectrometer (3000 V capillary voltage, 70 V fragment voltage) was connected. Mobile phases contained 60–70% (*v*/*v*) water with 0.065% trifluoroacetic acid and 30–40% (*v*/*v*) acetonitrile with 0.05% trifluoroacetic acid. Targeted peptides were quantified via calibration curves prepared from HPLC-MS chromatogram peak areas (mAU×s or Abundance×s, respectively). The Limits of Detection (LODs) of this method were determined to be between 2.5 and 10 µg/mL. 

### 2.3. Safety Studies for LbL-CoV19 Formulation 

Two sets of safety studies for LbL-CoV19 formulations were performed at Charles River Labs. First, we determined the potential local and systemic toxicity of LbL-CoV19 when given by IM injection on Days 1 and 29 to CD^®^ Sprague Dawley rats, followed by a 4-week recovery period to evaluate the potential reversibility of any findings. The animals were obtained from Charles River Laboratories, Kingston, NY, USA. They were all approximately 10 weeks old. The weights for males were 283–381 g, and for females were 187–246 g. A supplemental diet was provided to the animals as warranted by clinical signs or other changes and the acclimation period was 7 days. The study design is provided in the Supplementary Material Table S1A. The following parameters and endpoints were evaluated in this study: mortality, clinical observations (animal behavioral and welfare), body weight changes, food consumption, body temperature, Draize scores, clinical pathology parameters (hematology, coagulation, clinical chemistry, and urinalysis), plasma and tissue qPCR biodistribution analysis, organ weights, and macroscopic and microscopic examinations. 

Second, we performed a maximum tolerated dose and repeat-dose intramuscular injection study of LbL-CoV19 in rabbits. The animals were obtained from Envigo Global Services Inc., Denver, PA, USA. The rabbits were all approximately 7.5–8 months old. This study was performed in two phases. In Phase A, the weight of male rabbits was between 3.2 and 3.4 kg and 2.8 and 3.9 kg for females. In Phase B, the weight of males was between 3.2 and 3.5 kg and 2.6 and 3.5 kg for females. A supplemental diet was provided to the animals as warranted by clinical signs or other changes and the acclimation period was 7 days. The study design is provided in Supplementary Material Table S1B. The following parameters and endpoints were evaluated in this study: mortality, clinical observations, body weight data, food consumption, and anti-drug antibody evaluation.

The raw data for each endpoint, sex, and group were statistically evaluated and compared to controls. These groups were analyzed using an overall one-way ANOVA F-test if Levene’s test is not significant or the Kruskal–Wallis test if it is significant. If the overall F-test or Kruskal–Wallis test is found to be significant, then pairwise comparisons were conducted using Dunnett’s or Dunn’s test, respectively. Results of all pair-wise comparisons were reported at the 0.05 and 0.01 significance levels. All endpoints were analyzed using two-tailed tests unless indicated otherwise. 

### 2.4. Generation of HIS-CD4/CD8 Mice

HIS mice were generated using a previously published method [16]. In general, three-week-old NSG-B2m mice were administrated intravenously (IV) adeno-associated virus serotype 9 (AAV9) which encodes human IL-3, IL-15, and GM-CSF. The animals were then perithoracically injected with AAV9-encoding HLA-A2 (HLA-A*0201), HLA-DR1 (HLA-DRB1*01) and HLA-DR4 (HLA-DRB1*0401). After two weeks, a 150 Gy whole-body sublethal irradiation was administrated to all the mice. Several hours later, each mouse was engrafted with 1 × 10^5^ CD34+ human HSCs via IV. CD34+ human HSCs were isolated from HLA-A2, HLA-DR1, and HLA-DR4-matched cord blood samples. 

### 2.5. Immunogenicity Studies for LbL-CoV19 Formulations

Immunogenicity testing was performed using either NSP peptide 22 (RdR polymerase, LMIERFVSL), M peptide (NRFLYIIKL), N peptide (ASWFTALTQHGK), ORF3a (EPIYDEPTTTTSVPL, and CD4+T cell epitope) and/or Spike protein in LbL-CoV19 formulations (LbL-CoV-1, 2, 3, 4, and 5), as shown in Table 1. The immunogenicity studies were performed via intramuscular injection in HIS mice, with 5 mice per group and one vaccine dosing per group.

- IFN-γ-ELISpot assay

The relative numbers of splenic COVID-19 Spike protein, NSP peptide 22 (RdR polymerase, LMIERFVSL), M peptide (NRFLYIIKL) and N peptide (ASWFTALTQHGK), as well as ORF3a (EPIYDEPTTTTSVPL, CD4+T cell epitope)-specific, IFN-γ-secreting human CD8+ and CD4+T cells of COVID-19 vaccine-immunized mice were determined by a human IFN-γ ELISpot^PRO^ kit (Mabtech AB, Stockholm, Sweden) as described previously [15]. Briefly, 12 days after the immunization with COVID-19 vaccines, splenocytes were isolated from the spleens harvested from the mice. In each well of the ELISpot 96-well plate, 5 × 10^5^ splenocytes in culture medium (RPMI-1640, 10% FBS serum, antibiotics, and 5 × 10^−5^ M of 2-mercaptoethanol) were placed. The plate was pre-coated with anti-human IFN-γ monoclonal antibody (1-D1K). A concentration of 1 μg/mL of respective peptide was added to the plate and then incubated for 24 h (37 °C with 5% CO_2_). The plates were washed in PBS 5 times. A volume of 100 µL of a horseradish peroxidase-conjugated anti-human IFN-γ detection monoclonal antibody (7-B6-HRP) was added and incubated for 2 h at RT. An amount of 100 µL/well of TMB solution was added after five more washes and incubated for 10 min. The number of spots in each well were counted. IFN-γ-secreting CD8+ T cells were analyzed, and a “Stimulation Index” was calculated (spots detected in the respective peptide/spots in the media).

- ELISA for serum evaluation

Opaque microtiter plates (96-well; Thermo Fisher, Waltham, MA, USA #15042) were coated with 1 µg/mL of the Human Angiotensin-Converting Enzyme 2-Fc chimeric protein (50 µL/well) (GenScript #Z03516). Following overnight incubation at 4 °C, plates were washed 4 times with 1× PBS/0.05% Tween-20 and blocked for 1.5 h at 37 °C (SuperBlock- #37515, Blocking Buffer, Thermo Fisher, Waltham, MA, USA). After washing, dilutions of the His-tagged SARS-CoV-2 Spike protein as a positive control (Genscript # Z03483) corresponding to the receptor binding domain of virus or spike protein sample released from the NPs was added (1× PBS with 0.05% Tween-20). Plates were incubated for 2 h at 37 °C and washed with 1× PBS with 0.05% Tween-20. HRP-conjugated rabbit anti-His (GenScript # A00170-40) was added at a 1:20,000 dilution in assay diluent (100 µL/well). Plates were incubated for 1 h at 37 °C, washed as previously described, and then bound antibody was detected with Super Signal ELISA Pico Chemiluminescent substrate (Thermo Fisher # 37070). Super Signal substrate reagent was prepared according to manufacturers’ directions (50% Luminol-Enhancer Substrate + 50% Stable Peroxide solution, Thermo Fisher, Waltham, MA, USA) and 100 µL was added to each well. The plate was then inspected for luminescence on a Microplate Reader (BioTek Synergy HTX Multi-Mode, BioTek, Winooski, VT, USA). The positive control was the SARS-CoV-2 Spike protein at a known concentration range, and the negative control was incubated in the block buffer without the SARS-CoV-2 Spike protein.

- Human ICCS assay

ICCS assays were performed as previously described [15]. Briefly, in the presence of brefeldin at 37 °C with 5% CO_2,_ the splenocytes isolated from the LbL-CoV19 vaccine-immunized HIS-CD4/CD8 mice were stimulated for 4–6 h using a pool of the synthetic peptides. The lymphocytes were then blocked by anti-mouse CD16/CD32 antibody and stained with surface biomarkers (Pacific Blue anti-human CD45, clone HI30l; PE-Texas Red anti-human CD3, clone UCHT1; APC-Cy7 anti-human CD4, clone RPA-T4; and FITC anti-human CD8, clone HIT8a). Lymphocytes were then permeabilized, labeled with the FITC-anti-human IFN-γ antibody, and fixed with 1% paraformaldehyde for flow cytometry analysis (BD LSR II, BD Biosciences, Franklin Lakes, NJ, USA).

### 2.6. Efficacy Study 

For the SARS-CoV-2 challenge model, we used a virus strain BA1 for challenging HLA-A2 transgenic (Tg) B6 mice [17]. To confirm the pathogenic potential of SARS-CoV-2 BA1, mice (Strain #: 003475, Jackson Laboratories, Bar Harbor, ME, USA) were infected with either saline (mock), or 1 × 10^5^ plaque-forming units (PFU) of the SARS-CoV-2 BA1 first to determine the virus challenge dose. The SARS-CoV-2 BA1 virus was obtained from BEI (cat # NR-55329). All work with infectious SARS-CoV-2 was performed in approved BSL3 facilities at Columbia University Irving Medical Center using appropriate positive-pressure air respirators and protective equipment. The Median Tissue Culture Infectious Dose (TCID50) assay was used to evaluate the virus load. Lung homogenates of challenged mice were collected in the approved BSL3 facility for setting up the TCID50 titration assay. Serial 2-fold dilutions of the samples were added to a 96-well plate seeded with 2 × 10^4^ Vero-E6 cells (ATCC #1586) at 37 °C in Dulbecco’s Modified Eagle medium (DMEM) supplemented with 10% fetal bovine serum (FBS), 10 mM HEPES pH 7.4, and 100 U/mL of penicillin–streptomycin under 5% CO_2_. Three days later, the wells were visually scored for cytopathic effect (CPE, as observed by light microscopy) of the cells at each dilution, to determine the endpoint TCID. For efficacy studies, 5 groups (4 formulations and 1 control) with 6 mice (7–8-week-old) per group (30 total animals) were used. The final four vaccine formulations, LbL-NSP peptide 22 (RdR polymerase, LMIERFVSL)-spike, LbL-NSP peptide 22 (RdR polymerase, LMIERFVSL) + adjuvant-spike, LbL-ORF3a (EPIYDEPTTTTSVPL, CD4+T cell epitope)-spike, and LbL-ORF3a (EPIYDEPTTTTSVPL, CD4+T cell epitope) + adjuvant-spike, were referred to as LbL-CoV-1, 1a, 4, 4a, as shown in Table 1. Mice received two doses of a respective vaccine formulation intramuscularly every 3 weeks. Four weeks after the boosting and hACE2 gene transduction by AAV9, the mice were intranasally challenged with a determined effective dose of 10^5^ PFU BA1 variant. The next day, the mice were weighed and then observed daily for signs of illness and mortality. Four days later, sera were collected, and lungs were harvested and used for the determination of the amount of viral RNA by qRT-PCR. Live virus titer was also determined by using the tissue-culture infectious dose (TCID50) titration assay. 

### 2.7. Statistic Studies in Immunogenicity and Efficacy Tests

All statistical analyses were performed using GraphPad Prism (ver. 7) (GraphPad Software, Inc., La Jolla, CA, USA). Statistical Analyses at *p*-values of <0.01 (**); <0.001 (***); <0.0001 (****) statistically significant differences were established between the control and vaccine groups or between vaccine groups. An unpaired *t*-test was used to determine the differences between the two groups in the immunogenicity and efficacy studies. 

## 3. Results

### 3.1. LbL-CoV19 Vaccine Platform Synthesis Using a Microfluidic Device

- TMC nanoparticle synthesis using a microfluidic device

**Figure 2:**
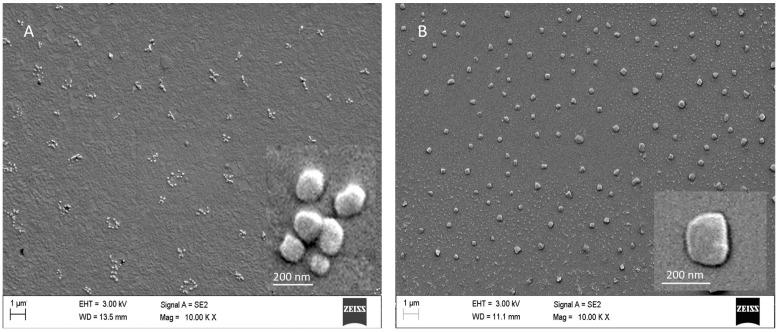
Scanning electron microscopy images of TMC nanoparticles (**A**) and LbL-CoV-1 formulation nanoparticles (**B**). The inserts in the right bottom corner images are high-magnification images for TMC nanoparticles and LbL-CoV-1 formulation nanoparticles.

To develop a scalable production vaccine platform, we synthesized the trimethylated chitosan TMC NP within the microchannels of a microfluidic device that utilizes continuous flow to obtain the final product. The device was designed to generate droplets for particle synthesis. When the positively charged TMC and the negatively charged TPP solution were mixed in the NanoGenerator, the continuous flow enabled a controlled droplet break-up, which is required for yielding monodisperse nanoparticles via electrostatic assembly. To accomplish this, we transitioned from the conventional mixing method, which was described previously [13], to a lab-scale microfluidic device (NanoGenerator Flex Nanoparticle Synthesis system, PG-SYN-8, PreciGenome, San Jose, CA, USA Figure 2). We could obtain 10 mL production in less than 10 min using the microfluidic device, but using the conventional method, we could only obtain 1 mL in 1 h. For further scale-up, we could obtain 1 L production using a larger model of the reactor (NanoGenerator Max, PreciGenome, San Jose, CA, USA). 

During initial experiments, pre-set pressures from a pressure controller were applied to Reservoirs 1 and 2 in a microfluidic device. Solutions in each reservoir were pushed through tubing into the two inlets of a microfluidic chip and mixed inside the channel of the microfluidic chip. The nanoparticle product solution could be collected from the outlet of the microfluidic chip after a few seconds to less than 10 min. The flow rate of Reservoir 1 could be tuned from 0 to 10 mL/min while the Reservoir 2 flow rate could be tuned from 0 to 1 mL/min in the current model of the reactor. As a result, the main variables that can be tuned are the two precursor flow rates and concentrations of input chemistries during the synthesis.

To initiate the production of TMC nanoparticles using the PreciGenome system, we first adapted the same concentration of precursors in the conventional method to enable direct comparisons between methods. We achieved better repeatability (three repeats), a narrower size range of NPs (180–210 nm) and a small Polydispersity Index (PDI) of NPs. After 24 h of storage under refrigeration, the NPs did not demonstrate significant changes in average size (via DLS) or Zeta potential value, which further confirmed the NPs were successfully produced and remained stable under storage (Table 2). 

Next, the input concentrations of precursors (TMC and TPP) were tuned to investigate their effect on NP synthesis. We investigated concentrations between 2 and 5 mg/mL for TMC and/or TPP. To obtain consistent results and good-quality NPs, we determined a concentration of 2–2.5 mg/mL for TMC was optimal. We then kept the concentration of TMC at 2 mg/mL but tuned the flow rate for the reactions. When the flow rate was either lower than 6 mL/min or higher than 9 mL/min, nanoparticles formed with a wider size distribution or with multiple size ranges. It was determined that the optimal total flow rate needs to be controlled between 7.7 mL/min and 8.8 mL/min, and the individual concentration of precursor TPP set at 4 mg/mL. If the TPP concentration is decreased to 2 mg/mL, the best total flow rate would be 6 mL/min (grey highlights in Supplemental Material Table S2) to be able to obtain a single-size distribution of nanoparticles. The average size from DLS for these conditions is between 380 and 470 nm, which is larger than the NPs made using lower concentrations or flow rates. However, the results were very repeatable and indicated that both flow rates and precursor concentrations are key for controlling the formation of nanoparticles. When precursor concentrations were too low, there would be the formation of many small nanoparticles. To increase the NP density or yield, or increase the size of the NPs, the concentration of precursors needs to be increased accordingly. 

After finalizing the NP formation conditions, we evaluated the repeatability of the reaction between separate batches. Three repeated reactions were performed for this study. TMC and TPP were separately dissolved in ultrapure DI water at a concentration of 2 mg/mL for both. The final flow rate of TMC was 5 mL/min and of TPP was 1 mL/min which attributed to the total flow rate of 6 mL/min. The consumed TMC solution was 1 mL, the consumed TPP solution was 0.2 mL for each reaction, the ratio of TMC and TPP was kept at 5:1, and the total obtained TMC nanoparticle solution was approximately 1.2 mL. The reaction time was 0.2 min (12 s) for each batch. The average size of the resultant TMC-TPP NPs was 206.3 ± 18.9 nm (DLS measurement), which was within our targeted size range of 200–400 nm. Following production, we lyophilized the NP solution to generate a powder after freeze drying overnight. The yield of NP was 93–97% after weighting from lyophilized powder. We also determined that the first obtained batch had a higher variation in sizes compared to subsequent production batches. The main reason is the microfluidic tubes were not filled with precursors at the start of the first batch production. To achieve consistent production, the first batch needs to be eliminated, or the precursor should be flowed through the tubing for at least 30 s to allow filling with both precursors. 

In the SEM measurement of the produced TMC NPs, we determined that the NPs were uniform in size at a range of 66.2 ± 13.5 nm, as shown in Figure 2A. These sizes are much smaller than what we obtained from the DLS measurement, which is as expected. DLS provides the sizes of NPs corresponding to the hydrodynamic diameter, which is usually larger than the size of the core provided by SEM (without hydration or solvation layer). SEM imaging indicates a 2D representation of a 3D nanoparticle. SEM looks at the electron-dense part of a particle and for each particle individually. Additionally, SEM informs us about the nanoparticle’s morphology, showing a quasi-spherical shape for TMC nanoparticles.

- LbL-CoV19 vaccine formulation synthesis by microfluidic device

After confirmation that we could obtain the high-quality and repeatable batch-to-batch production of TMC NPs from the microfluidic reactor, we next developed the method for the synthesis of vaccine formulations. To encapsulate antigen subunit proteins or peptides in TMC nanoparticles, we premixed protein/peptide with either TMC or TPP depending on the isoelectric point (pI) value of proteins or peptides and then loaded the solutions in the reactor reservoir. If the PI value of the antigen was higher than pH 7 (reaction solution pH), the antigen had to be premixed with TMC; otherwise, we premixed it with the TPP solution to allow attachment between peptide/protein to precursors. From the conventional method to microfluidic synthesis, we found that the DLS sizes of TMC–antigen–TPP NPs obtained were different. Compared to prior work using the conventional production method, the size of NPs was smaller (200–300 nm) using the microfluidic production system than it was in the original batch-to-batch emulsion method (300–400 nm). Further, the standard deviation was also decreased from 163 nm to 32 nm for TMC–antigen–TPP (LbL-CoV19) nanoparticle synthesis and from 63 nm to 19 nm for TMC nanoparticle synthesis when using the microfluidic reactor. This is primarily due to the high-speed mixing of precursors using microfluidic devices and the increased control over production parameters. This method of production will likely be feasible for future GMP production. In the SEM results (Figure 2B), we observed that LbL-CoV19 formulation nanoparticles were 94.6 ± 27.7 nm in diameter, generated from 60 nanoparticles. After the loading of antigen proteins, the shape of NPs was changed not to be perfectly spherical or quasi-spherical, which contributed to the difference found between DLS and SEM. As a result, we could use both methods to evaluate LbL-CoV19 formulation to provide a comprehensive consideration of the quality of NP formulations. 

### 3.2. In Vitro Release of Antigens on LbL-CoV19 Formulations

We next developed the method for production of a two-layer formulation, which included both peptide and spike protein in one LbL-CoV19 formulation. In this study, we used two methods for antigen loading. The first was a one-step synthesis (1-step) without purification between loading of each layer of antigen (Spike and peptide 22), and the second was a two-step synthesis (2-step) with purification performed before adding the second layer of Spike protein. In vitro release tests were performed to determine which method would be best for vaccine candidate preparation.

**Figure 3:**
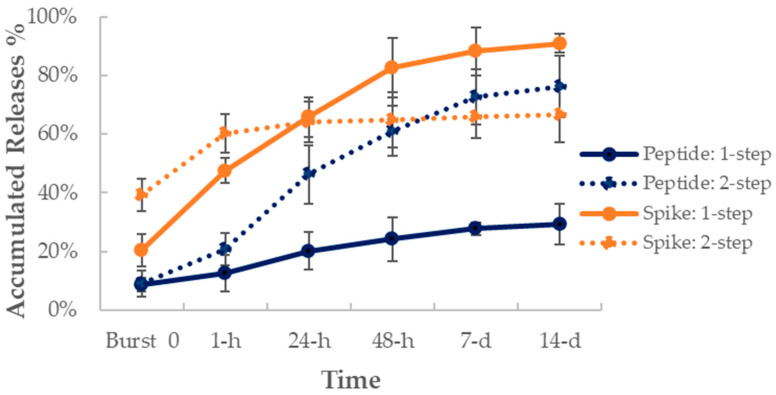
NSP Peptide 22 and Spike protein (SP) release profiles over two weeks from 1-step (solid line) and 2-step (dot line) synthesized two-layer LbL-CoV19 vaccine formulation (LbL-CoV-1). Mean (dots) ± SEM is represented for each of the above graphs.

After loading the two layers of antigens in a layer-by-layer fashion, we investigated the release profile in an in vitro setup. Peptides were evaluated and quantified through the HPLC-MS method. The results in Figure 3 demonstrate that after adding a purification step between antigen loading stages (2-step, blue dot line), the release rate for peptide increased by almost 2x as compared to the one generated from one-step synthesis without purification (1-step, blue solid line). The 1-step release of the peptide was much slower, which allowed about 30% of the peptide to be released in two weeks. One potential explanation for this behavior is that the addition of a purification step during the two-antigen loading process interrupted the peptide encapsulation process, as a result, the peptide is pushed out of the TMC NP core structure of the nanoparticles during the purification process. Furthermore, the loading efficiency for 1-step was 91.4–93.7%. After the additional step of purification, the loading efficiency was decreased to 79.0–84.9% which indicates a large quantity of peptide loss due to the additional purification step. 

In this same release test setup, we calculated the released Spike protein amount at each collection time point. We used a standard curve that was generated from the ELISA method to determine the released amount. Most loaded spike protein was released in the first 48 h, as shown in Figure 3 for the 1-step synthesis method (orange solid line). At 48 h, approximately 83% of the total Spike protein was released from the NPs. The released dosage in two days potentially provides enough doses of antigens as a vaccine to stimulate a humoral immune response. For the 2-step synthesis method (Figure 3, orange dotted line), we found almost 40% of spike protein was found in the burst release, and then another 20% of the protein was released in the first 1 h; after 24 h, the release rate was slower. When compared with peptide release, the spike protein was released first from the outer layer of the NPs, and the core peptide was released slowly out of the NPs over a relatively longer period. From these results, we concluded that the use of 2-step synthesis is not an optimal condition for two-antigen loading and delivery. We therefore selected the 1-step synthesis method for vaccine candidate production to use in the animal studies. 

### 3.3. Method Developed for Reconstitution of Lyophilized Vaccine Formulation

**Figure 4:**
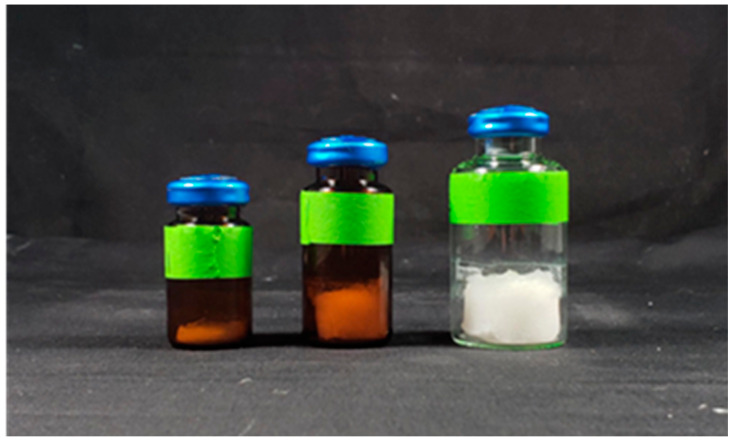
The lbL-CoV19 vaccine candidate was filled in an amber bottle with different scales/doses by lyophilization and then sealed with a rubber stopper and crimped with an aluminum cap to be ready for reconstitution using saline.

We next investigated the reconstitution of lyophilized LbL vaccine formulations that included the two antigens (peptide inside core, spike protein loaded on outer layer). After synthesis, we lyophilized the formulation solution for 48 h, and a pure white lyophilized powder was obtained as shown in Figure 4. Following lyophilization the vial is sealed with a rubber stopper and crimped with an aluminum cap to be ready for reconstitution using saline at the point of use. We used DLS to evaluate and track the size changes in solution before and after lyophilization, and then after reconstitution into solution in a time course. The NPs were redispersed by adding to a saline solution to and vortexing for 1, 5, 10, and 20 min. We tested shaking, bath sonication, and vortexing as the three methods for reconstitution. During the reconstitution process, we monitored the changes in size via DLS. We determined that after vortexing for 10–20 min, the NPs are well redispersed in solution without significant changes in NP size. This result indicates that the formulation could be stabilized and reconstituted easily before vaccination. However, additional work will be required to develop a vaccine formulation that can be rapidly resuspended (less than 1 min) at the point of vaccination, potentially by including additional suspension additives like starches.

### 3.4. Demonstrating the Safety Profile of LbL-CoV19 Vaccine Candidates 

To develop a safe vaccine candidate and determine appropriate dosing, we investigated local and systemic toxicity of the layer-by-layer COVID19 vaccine candidate (LbL-CoV19), when given by intramuscular (IM) injection on Days 1 and 29 to CD^®^ Sprague Dawley rats. We also evaluated the potential reversibility of any adverse events after a 4-week recovery period (GLP study). LbL-CoV19-related clinical pathology findings included mildly higher aspartate aminotransferase and/or alanine aminotransferase activities at dosings ≥ 50 µg/day, mildly higher fibrinogen concentrations at dosgings ≥ 10 µg/day, and/or minimally lower albumin concentrations at dosings ≥ 50 µg/day in both sexes. The dosings described here only indicate the amount of antigen used and do not include the mass of NPs. The mass ratio of NP to antigen is 6:1. These findings are considered consistent with an acute phase response. Males at dosings of 100 µg/day and females at greater than ≥50 µg/day all had minimally to mildly higher potassium concentrations. All differences in clinical pathology parameters associated with LbL-CoV19 resolved after the 26-day recovery period. Based on the initial findings, there were no LbL-CoV19-related mortalities or LbL-CoV19-related effects on clinical observations, body weight, food consumption, body temperature, Draize scores, hematology, urinalysis, organ weights, or other macroscopic observations.

LbL-CoV19-related microscopic findings occurred in the injection sites and sciatic nerves in males and females at 100 µg/kg/day. These consisted of minimal to moderate myofiber degeneration/necrosis, minimal to moderate mixed cell inflammation, and minimal to mild hemorrhage in the right (Day 29) injection site in males and females; minimal myofiber degeneration and necrosis in the left (Day 1) injection sites in males and females; minimal perivascular mixed cell infiltrates, mononuclear cell infiltrates, fibroplasia in the surrounding adipose tissue in females. The decrease in incidence and severity of the findings in the injection site and sciatic nerve on the left side compared to the right indicates partial recovery of these findings over the terminal period of 31 days.

Intramuscular administration of LbL-CoV19 once every 4 weeks (Days 1 and 29) was systemically tolerated by CD^®^ Sprague Dawley rats up to the highest dose tested of 100 µg/kg/day. Local tolerability through the terminal interval was only noted up to 50 µg /kg/day due to the observation of necrosis, degeneration, hemorrhage, and inflammation at the dose sites and sciatic nerves. However, there were signs of recovery noted when comparing the tissues representative of Day 1 against Day 29. Therefore, the systemic no-observed-adverse-effect-level (NOAEL) was at the dose of 100 µg/kg/day, while the local no-observed-adverse-effect-level (NOAEL) was at the dose of 50 µg/kg/day. 

New Zealand White Crl: KBL(NZW) rabbits were then chosen to further evaluate the potential toxicity of LbL-CoV19 and determine the maximum tolerated dose when given by intramuscular injection, once on Day 1 and then weekly for at least two doses. For mortality, all animals survived to study termination. There were no LbL-CoV19-related clinical or veterinary signs observed. Any other noted clinical observations or skin reactions were sporadic, lacked a dose–response relationship, and/or were within the range of normal findings for group-housed animals of this age, sex, and species and were not considered test article-related. For evaluation of skin reaction, there were no positive Draize Scores observed. There also were no LbL-CoV19-related effects on body weight observed. Any other minor fluctuations among mean and individual body weight were considered sporadic, consistent with biologic variation, lacked a dose–response relationship, and/or negligible in magnitude and not test article-related. There were no LbL-CoV19-related effects on food consumption observed. Following either a single or double administration of LbL-CoV19 formulation at dose levels of 5, 10, 25, and 50 µg/kg/dose, results indicated all dose levels were well tolerated and there were no LbL-CoV19 related effects noted in any parameter examined. Dosings described here again only indicate the amount of antigen used and do not include the mass of NPs. These results provide information for dose design in immunogenicity and clinical studies.

### 3.5. Demonstration of Immunogenicity and Efficacy of COVID-19 Vaccine 

**Figure 5:**
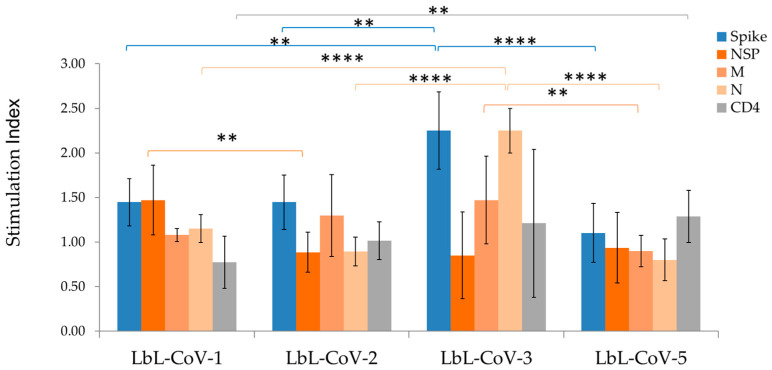
ELISpot immunogenicity studies for LbL-CoV19 formulations. The number of IFN-γ-secreting cells was measured by ELISpot assay. The stimulation index was calculated as the number of spots detected in the respective peptide stimulated well divided by the number of spots in the media-only well, and the bars displayed are the mean of 5 animals per dose group. Samples were run in duplicate. **** indicates *p* < 0.0001; ** indicates *p* < 0.05 significant between two groups of vaccine formulations.

For evaluating the immunogenicity of the LbL-CoV19 vaccine candidate, we used a one-layer formulation that included only the spike protein (LbL-CoV-5, Table 1) and a two-layer formulation with both the Spike and T cell epitope peptide (LbL-CoV-1, 2, 3 formulations). From the ELISpot-T cell immunogenicity studies, we observed that all LbL-Spike vaccines (LbL-CoV-5) induced CD8+ T-cell response, secreting IFN-γ against Spike peptide, VVFLHVTYV, which is known to be restricted to HLA-A2. Among those, LbL-CoV-3 (LbL-N peptide-Spike) including the N peptide in the formulation that is restricted to HLA-DR1 induced the highest degree of response (Figure 5) in all the formulations. The LbL-CoV-3 vaccine also induced a high level of N peptide-specific CD4+ T cell response. Similarly, LbL delivery of the Spike protein and encapsulated with NSP RdR polymerase #22 peptide vaccine (LbL-CoV-1, LMIERFVSL: restricted to HLA-A2 [18]) and LbL-Spike with the M peptide vaccine (LbL-CoV-2, NRFLYIIKL: restricted to HLA-C07) induced spike peptide-specific and M peptide-specific CD8+ T-cell responses, respectively.

**Figure 6:**
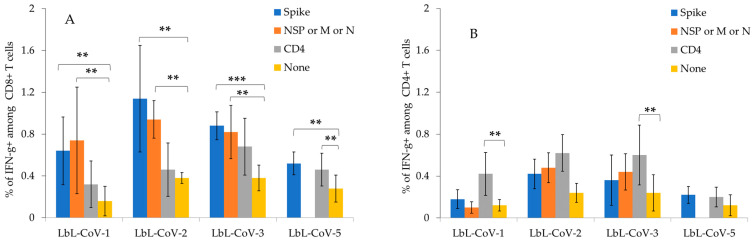
ICS studies of human CD8+ T cells (**A**) and CD4+ T cells (**B**) induced by the LbL-CoV19 vaccine candidates as determined by single cell-based multiplexed assay. The bar displays each mean ± SEM for each of the above graphs. *** indicates *p* < 0.005, ** indicates *p* < 0.05 significant between each group to control.

In the intracellular cytokine staining (ICS) assay, we found that all LbL-CoV19 vaccine formulations induced CD8+ T cells secreting IFN-γ against Spike #29 peptide. LbL-Spike with RdR polymerase #22 peptide vaccine candidate (LbL-CoV-1) and LbL-Spike with M peptide vaccine candidate (LbL-CoV-2) induced Spike #29 peptide-specific and M peptide-specific CD8+ T-cell responses, respectively. LbL-Spike with N peptide (LbL-CoV-3) vaccine candidate induced not only N-peptide-specific and CD4 peptide-specific CD4+ T cells that secrete IFN-γ, but also N-peptide-specific and CD4 peptide-specific CD8+ T cells that secrete IFN-γ. This is likely due to a bystander CD8+ T-cell activation via activated CD4+ T cells. In this regard, it is plausible that some of the T cells secreting IFN-γ induced by LbL-Spike with the N peptide vaccine, as identified in the ELISpot assay, could be not only CD4+ T cells but also include CD8+ T cell population (Figure 6A,B). As for CD4+ T cells, we could successfully identify CD4 peptide-specific CD4+ T cells secreting IFN-γ with all LbL-Spike vaccines by ICS.

**Figure 7:**
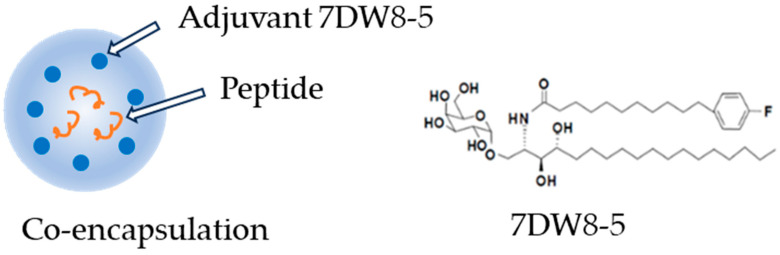
Co-encapsulation of adjuvant 7DW8-5 with peptide in formulation LbL-CoV-1.

Upon the preliminary result, we further compared LbL-CoV19 formulations with those that included the T-cell enhancer/adjuvant 7DW8-5 within the core of formulations to serve as other candidates in the immunogenicity studies. The schematic in Figure 7 illustrates the co-encapsulation of adjuvants and peptides inside the core of the LbL- CoV19 formulation. Only one formulation LbL-CoV-1 (LbL-pep22-Spike) was prepared for this set of studies.

**Figure 8:**
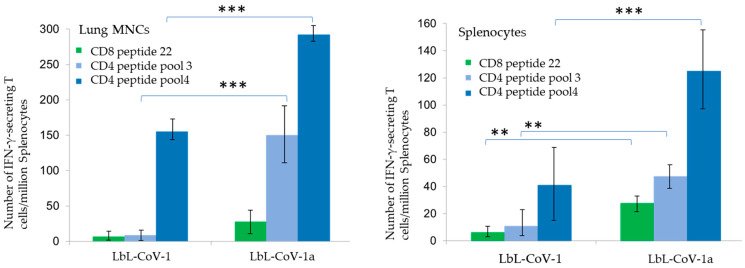
ELISpot of immunized mice CD4+ and CD8+ T cell responses from one dose of LbL peptide Spike formulation with (LbL-CoV-1a) and without (LbL-CoV-1) adjuvant 7DW8-5. The bar displays each mean ± SEM for each of the above graphs. *** indicates *p* < 0.005, ** indicates *p* < 0.05 significant between two group of vaccine candidate.

After administration in HIS mice, we found that both formulations including RdRp NSP peptide 22 and spike protein demonstrated CD 4+ and CD8+ T cell responses, but the two-layer formulation with adjuvant (LbL-CoV-1a) generated much higher responses, as shown in Figure 8. It was confirmed that the NSP peptide is a much stronger binder to HLA-2 and an immunodominant peptide. Further, the peptide encapsulated with the 7DW8-5 adjuvant stimulated an even higher CD8+ T cell response than that observed for the peptide formulation alone (LbL-CoV-1). Encouragingly, both formulations generated either CD8+ or CD4+ responses in splenic and lung-resident T-cells in mice after only one dose. 

In ELISA tests with serum collected from mice, we found that after the IM administration of vaccine candidates, all the test vaccine formulations presented humoral responses. However, the two-layer formulation including both spike and NSP peptide (LbL-CoV-1) demonstrated a much higher response (2.4–5.6 times higher) as compared with the one-layer formulation including only the spike protein (LbL-CoV-5). We also analyzed serum obtained from rabbits used as described in the safety study Section 3.3. An electrochemiluminescence ligand binding assay was developed and subsequently used to detect the titer of anti-Spike protein antibodies in rabbit serum samples after one or two doses delivered intramuscularly (evaluated at Charles River Lab, Wilmington, DE, USA. A total of 26 samples showed the positive presence of these antibodies, all from samples obtained 7 or 14 days after single or double dose administration. The titers ranged from 1.05 to 556, which indicated high immune responses were stimulated in only 7–14 days after vaccination in rabbits. 

### 3.6. SARS-CoV-2 Challenge Studies

**Figure 9:**
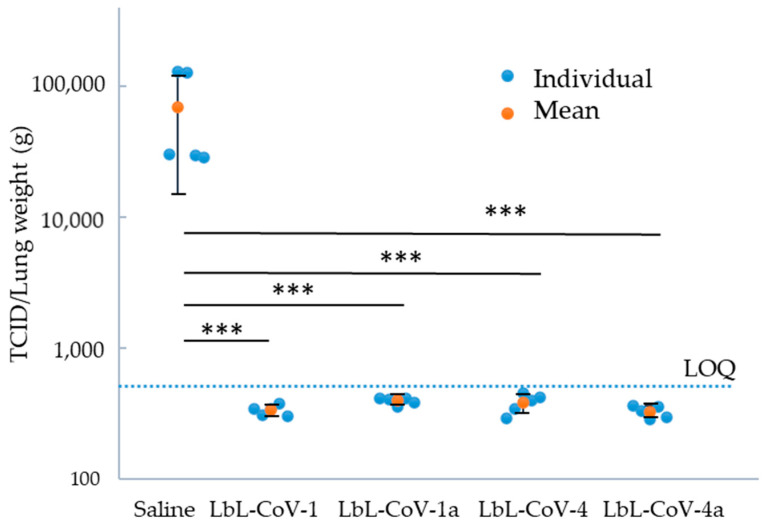
Virus MA 10 strain TCID50 titers were determined in Lung tissue after 3 days of viral challenge for the saline mice group and four LbL-CoV1, 1a, 4, 4a vaccine groups. Each dot data point represented a mouse (blue) and an average group of mice (red) dot with STDEV of log TCID/lung weight. The dotted line indicates the limit of quantitation of the viral load. *** indicates *p* < 0.001 significant between each group to saline control.

To confirm the BA1 virus could infect immunodeficient mice, we first performed the virus BA1 dose determination. Three days post-injection it was notable that infection with 10^2^ PFU of SARS-CoV-2 produced increased weight loss. In lung tissue/cells, high levels of virus load were found. As a result, we confirmed that the BA1 virus strain can infect transgenic mice, and this was eventually used for the efficacy studies. As shown in Figure 9, four LbL-CoV19 formulations tested here were demonstrated to significantly decrease virus load to an undetectable level from mice infected after three days of viral challenges. Although we did not see differences between each formulation group, compared to non-vaccinated groups, the viral load for all vaccinated groups was observed to decrease significantly below the limit of quantification (LOQ). The lung weight of the saline group (at an average of 104 mg) was also demonstrated to be significantly lower than the four vaccinated groups’ animals three days post-infection (127–154 mg). We found that the LbL-CoV19 vaccines can rapidly protect mice from infection. In potential upcoming studies, we will continue evaluating the formulations at lower doses and evaluate long-term broad protection using challenges with multiple variants to evaluate the potential full protections. 

## 4. Discussion

A conventional method for TMC nanoparticle and LbL-vaccine formulation synthesis was previously developed [13]. The technique utilizes a batch-wise system and the principle of dropwise addition of the cross-linker molecules such as TPP, and/or the antigens such as proteins or peptides, to the trimethylated chitosan solution. However, conventional techniques require qualified personnel throughout the production process, resulting in a non-controllable batch-to-batch variation even at a very small scale. Therefore, this previously developed batch-wise method is difficult to transfer to a Good Manufacturing Practice (GMP) process. We therefore developed a method to overcome these issues by synthesizing the nanoparticle formulations within the microchannels of a microfluidic device that utilizes continuous flow to obtain the final product. To accomplish this, we transitioned the production to a lab-scale microfluidic NanoGenerator device). In the microfluidic system described in the experimental section, we obtained 10 mL production in less than 10 min, while using the conventional method we only obtained 1 mL in 1 h. Yield could easily be increased to 1 L production using another model of the reactor (NanoGenerator Max, PreciGenome, San Jose, CA, USA). 

The device was designed to generate droplets for nanoparticle synthesis. The continuous flow enables a controlled droplet break-up, which is required for yielding monodisperse nanoparticles. In the reaction, the positively charged trimethylated chitosan (TMC) and the negatively charged TPP solution were mixed in the NanoGenerator, and NPs were formed via electrostatic assembly. This method reduces the reaction time while better controlling the final NP composition, narrowing the size distribution, and increasing the repeatability of synthesis with the potential for large-scale vaccine production. We also determined that the flow pressure and flow rate for each precursor solution influence the size distribution of the final NP formation. As a result, the size and surface charge of the TMC nanoparticles can be more easily controlled and tuned for different layers of antigen or adjuvant loading purposes using the developed microfluidic synthesis approach. The developed production method demonstrated batch-to-batch reproducibility and is ready for transfer to GMP production. 

Several factors influence drug release from TMC NPs, including the composition, composition ratio, ingredient interactions, absorbed protein properties, preparation methods, etc. [19]. In this study, a small peptide and/or adjuvant was encapsulated inside the core of TMC NPs, and the spike protein was adsorbed on the outer polymer layer for dual/two-phase antigen delivery. We achieved greater than 80% antigen loading efficiency for both layers. From the experiment prediction, the layer-by-layer release of the two antigens was demonstrated. The release profile reveals that the spike protein was delivered first by diffusion from the surface of the nanoparticle. Since the outside layer spike protein is non-covalently complexed within the polymer matrix, it was released first once administrated by the IM route. The observed burst release of 10–15% of the outer layer spike protein can be hypothesized for immediate humoral antibody generation and protection since the released spike proteins and the non-fully released formulations were taken up by immune cells, mostly through ACE-2 receptor-mediated endocytosis and activated host immunity as the first protection. The continued releases of core peptides/adjuvants due to the rupture of the core by swelling, osmotic pressure, and eventually degradation stimulated T-cell immunity for long-term protection. Peptide/adjuvants were delivered from within the TMC NP shell through the polymer matrix by the formation of nanopores in the shells or slow rupture of the shell. The rupture time is correlated with the crosslinker density. The slow release of peptides/adjuvants after the rupture reflects the high water uptake of the inner chitosan core and the beginning of swelling and dissolution, resulting in antigens diffusing into the external water phase. Increasing osmotic pressure, in addition to the molecular weight of chitosan being greater than that of antigens, and bulk degradation of the chitosan core itself resulted in an increased rate of antigen diffusion into the external water phase from the core. All these factors led to the demonstrated release kinetics. This system demonstrated a high initial release rate, followed by a decreased release rate related to the diffusion distance of the antigens with the solution medium [20]. The sustained release of spike protein facilitates longer-term protection against the virus. The later stage released peptide and adjuvant potentially stimulate T-cell response to achieve full protection. The TMC-NP delivery platform did not alter the nature of the antigens, as the released proteins or peptides were demonstrated to maintain their inherent antigenicity and stability, as determined through evaluation using multiple methods such as ELISA. In the immunogenicity studies, LbL-CoV19 formulation was used to immunize mice and demonstrated the generation of both CD8+ and CD4+ responses in splenic and lung-resident T-cells after only one dose via intramuscular administration. In a rabbit study of maximum tolerated dose via IM administration, all dosing levels and schedules were demonstrated as safe in rabbits. Further, after only 14 days, a rabbit serum antibody test demonstrated a very robust immune response generated from the two-dose IM-administered vaccine candidate. 

From both the previously reported works in the literature [21,22] and our research results [13], TMC-NP has shown excellent biocompatibility and low toxicity due to its chemical and structural similarity to the natural glycosaminoglycans. Chitosan is easily biodegraded into amino sugars that are harmless byproducts and absorbed completely in the body [23]. Furthermore, here, in a GLP safety study to determine potential local and systemic toxicity of vaccine formulations in Sprague Dawley rats, no formulation-related mortalities or clinical effects were observed after IM injection and a 4-week recovery period. This included no sign of concern for body weight, food consumption, body temperature, Draize scores, hematology, urinalysis, and organ weights. The systemic no-observed-adverse-effect-level (NOAEL) was determined to be 100 µg (antigen mass)/kg/day, while the local no-observed-adverse-effect-level (NOAEL) was 50 µg (vaccine mass)/kg/day. The investigation reported that chitosan nanoparticles or derivates do not generate any chronic toxicity in vivo animal models in a month [24]; however, continued investigation on chronic toxicity will be performed to eliminate the long-term safety concerns for applying delivery platforms in humans. In efficacy studies, vaccine candidate formulations were demonstrated to decrease the virus load to an undetectable level rapidly in lung tissue for all mice in each group (*n* = 5) three days post-viral infection, as compared with a saline group. Further, animal body weight and lung weight were shown to have no significant changes. This is an important milestone achieved from these preclinical studies. 

## 5. Conclusions

Immunization is one of the most cost-effective ways to improve health, save lives, and ensure long-term prosperity. Chitosan nanoparticles are classified as GRAS (generally recognized as safe) by the FDA [9]. Our LbL chitosan-based vaccine delivery platform is designed for the plug-and-play delivery of multiple antigens and adjuvants. It not only can deliver variants of virus spike proteins that are dominant in currently approved COVID-19 vaccine formulations but also enables the simultaneous and controlled delivery of peptides for T-cell epitopes or even other adjuvants in the same dose for potential long-term and broad protection. Chitosan NPs exhibit strong mucoadhesive properties [25], and thus they would have the potential as the delivery platform for mucosal/intranasal vaccine development. 

## 6. Patents

The technology described in this report is included in the non-provisional application PCT/US23/69681, “Layer-by-layer delivery of active agents”.

## Figures and Tables

**Table 1:** LbL-CoV19 formulations.

LbL-CoV19 Formulations	Core-Layer Antigen	Outside-Layer Antigen	Adjuvant
LbL-CoV-1	NSP peptide 22 (LMIERFVSL)	SP	no
LbL-CoV-1a	NSP peptide 22 (LMIERFVSL)	SP	yes
LbL-CoV-2	M peptide (NRFLYIIKL)	SP	no
LbL-CoV-3	N peptide (ASWFTALTQHGK)	SP	no
LbL-CoV-4	ORF3a (EPIYDEPTTTTSVPL)	SP	no
LbL-CoV-4a	ORF3a (EPIYDEPTTTTSVPL)	SP	yes
LbL-CoV-5	SP	N/A	no

**Table 2:** Three repeated empty TMC NP size and zeta potential data as synthesized using a microfluidic device and aged after 24 h.

TMC NP Sample No.	NP Average Size (nm)	Average Zeta Potential (mV)
1	180.8 ± 24.6	24.8 ± 10.1
1 (24 h)	150.4 ± 19.5	26.7 ± 6.8
2	175.2 ± 24.0	17.1 ± 9.7
2 (24 h)	173.2 ± 30.0	16.7 ± 4.7
3	189.3 ± 31.5	18.3 ± 9.4
3 (24 h)	205.0 ± 27.1	16.2 ± 6.5

## Data Availability

The data presented in this study are available on request from the corresponding author.

## Supplementary Materials

The following supporting information can be downloaded at: https://www.mdpi.com/article/10.3390/vaccines12030339/s1, Table S1A: A 4-week Study of LbL-CoV19 by intramuscular injection in rats with a 4-week recovery period (GLP); Table S1B: Maximum tolerated dose study for LbL-CoV-19 in rabbits (non-GLP). Table S2: Parameters of LbL nanoparticle synthesis using a microfluidic device.
