# Supplementary material for: Microfluidic Synthesis of Scalable Layer-by-Layer Multiple Antigen Nano-Delivery Platform for SARS-CoV-2 Vaccines
Source: Vaccines (Basel). 2024 Mar 21;12(3):339. doi: 10.3390/vaccines12030339 (PMC10975406; doi:10.3390/vaccines12030339)
Supplement: Supplementary file 1 [file vaccines-12-00339-s001.zip › vaccines-2886220-supplementary.pdf]

Supplementary Materials

Table S1A: A 4-Week Study of LbL-CoV19 by Intramuscular Injection in Rats with a 4-Week Recovery Period (GLP)

Group No.	Test Article	Dose Level (µg*/kg/day)	Dose Volume (mL/kg)	Dose Conc. (µg*/mL)	Main Study		Recovery Study	
					No. of Males	No. of Females	No. of Males	No. of Females
1	Control	0		0	10	10	6	6
2	LbL-CoV-19	10	0.2	50	10	10	6	6
3		50		250	10	10	6	6
4		100		500	10	10	6	6

Table S1B: Maximum tolerated dose study for LbL-CoV-19 in rabbits (non-GLP)

Phase A:

Group No.	Test Article	Dose Level (µg*/kg)	Dose Volume (mL/kg)	Dose Concentration (µg*/mL)	Main Study	
					No. of Males	No. of Females
1	Dose Level 1	5		20	1	1
2	Dose Level 2	10	0.25	40	1	1
3	Dose Level 3	25		100	1	1
4	Dose Level 4	50		200	1	1

[a] Based on most recent body weight measurement; * Based on antigen mass only.

Phase B:

Group No.	Test Article	Dose Level (µg*/kg/dose)	Dose Volume[a] (mL/kg)	Dose Concentration (µg*/mL)	Toxicokinetic Study	
					No. of Males	No. of Females
5	Control	0		0	2	2
6		5	0.25	20	2	2
7	Vaccine	25		100	2	2
8		50		200	2	2

[a] Based on most recent body weight measurement; * Based on antigen mass only.

Table S2: Title: Parameters of LbL nanoparticle synthesis using a microfluidic device.

Sample No.	TMC conc. (mg/mL)	TMC vol. (mL)	TPP conc. (mg/mL)	TPP vol. (mL)	TMC/ TPP ratio	Total vol (mL)	TMC flow (mL/ min)	TPP flow (mL/min)	Total flow (mL/ min)	Size peak (nm)	Average size (nm)
1	2	1	2	0.2	5:1	1.2	5	1	6	469.5	381
2	2	1	2	0.2	5:1	1.2	2.5	0.5	3	193, 22, 5	47
3	1	1	2	0.1	5:1	1.1	10	1	11	180.2, 12.3	102
4	2	1	4	0.1	5:1	1.1	10	1	11	209.1, 10.7	300
5	2	1.5	4	0.15	5:1	1.65	10	1	11	131.5, 13.7, 2979	27
6	2	1	4	0.1	5:1	1.1	8	0.8	8.8	520.1	395
7	2	1	4	0.1	5:1	1.1	9	0.9	9.9	160.6, 14.9, 4901	62
8	2	1	4	0.1	5:1	1.1	8.5	0.85	9.35	511.1, 5181	415
9	2	1	4	0.1	5:1	1.1	8	0.8	8.8	569.4	467
10	2	1	4	0.1	5:1	1.1	7.5	0.75	8.25	512.5	413
11	2	1	4	0.1	5:1	1.1	7	0.7	7.7	577.7	454
12	2	1	4	0.1	5:1	1.1	6	0.6	6.6	158.7, 8, 40.0	164
